# PANoptosis in autoimmune diseases interplay between apoptosis, necrosis, and pyroptosis

**DOI:** 10.3389/fimmu.2024.1502855

**Published:** 2024-10-31

**Authors:** Kangnan Liu, Mi Wang, Dongdong Li, Nguyen Truong Duc Duong, Yawei Liu, Junfu Ma, Kai Xin, Zipeng Zhou

**Affiliations:** ^1^ School of Osteopathy, Henan University of Chinese Medicine, Zhengzhou, China; ^2^ Rheumatology Department, The Third Affiliated Hospital of Henan University of Chinese Medicine, Zhengzhou, China; ^3^ Oncology Department, Henan Province Hospital of Chinese Medicine (The Second Affiliated Hospital of Henan University of Chinese Medicine), Zhengzhou, China; ^4^ Rheumatology Department, Henan Province Hospital of Chinese Medicine (The Second Affiliated Hospital of Henan University of Chinese Medicine), Zhengzhou, China

**Keywords:** PANoptosis, PANoptosome, apoptosis, necrosis, pyroptosis, autoimmune diseases

## Abstract

PANoptosis is a newly identified inflammatory programmed cell death (PCD) that involves the interplay of apoptosis, necrosis, and pyroptosis. However, its overall biological effects cannot be attributed to any one type of PCD alone. PANoptosis is regulated by a signaling cascade triggered by the recognition of pathogen-associated molecular patterns (PAMPs) and damage-associated molecular patterns (DAMPs) by various sensors. This triggers the assembly of the PANoptosome, which integrates key components from other PCD pathways via adapters and ultimately activates downstream execution molecules, resulting in cell death with necrotic, apoptotic, and pyroptotic features. Autoimmune diseases are characterized by reduced immune tolerance to self-antigens, leading to abnormal immune responses, often accompanied by systemic chronic inflammation. Consequently, PANoptosis, as a unique innate immune-inflammatory PCD pathway, has significant pathophysiological relevance to inflammation and autoimmunity. However, most previous research on PANoptosis has focused on tumors and infectious diseases, leaving its activation and role in autoimmune diseases unclear. This review briefly outlines the characteristics of PANoptosis and summarizes several newly identified PANoptosome complexes, their activation mechanisms, and key components. We also explored the dual role of PANoptosis in diseases and potential therapeutic approaches targeting PANoptosis. Additionally, we review the existing evidence for PANoptosis in several autoimmune diseases and explore the potential regulatory mechanisms involved.

## 1 Introduction

Programmed cell death (PCD) refers to a controlled, autonomous process mediated by various biomolecules to maintain intracellular homeostasis (1).Recently, researchers classified cell death based on morphological features, triggering mechanisms, and cellular context, identifying additional PCD types, including apoptosis, necrosis, autophagy, pyroptosis and cuproptosis (2, 3). PCD is crucial for regulating physiological and pathological processes, including tissue development, inflammation, and immune responses (4, 5). Earlier studies mainly focused on the mechanisms and functions of individual PCD types. However, increasing evidence suggests that PCDs do not operate independently but form a complex network of interactions (6). Recent studies have begun to uncover the interactions and crosstalk among the complex mechanisms of different PCDs (7). In 2019, Malireddi et al. (8) documented a novel innate immune-inflammatory PCD pathway called PANoptosis. PANoptosis is regulated by the PANoptosome complex, which involves crosstalk and collaboration among apoptosis, necrosis, and pyroptosis. However, the overall biological effects of PANoptosis cannot be attributed to any single type of PCD (9).

Autoimmune diseases are disorders characterized by a breakdown of immune tolerance to self-antigens, leading to abnormal immune responses that damage tissues or organs (10). These diseases often affect multiple organs and are incurable, severely impacting patients’ quality of life and imposing significant economic burdens on individuals and society (11, 12). The pathogenesis of these diseases is complex and involves multiple factors, with molecular mechanisms still not fully understood. Increasing evidence indicates that PCD plays a critical role in autoimmune diseases, particularly apoptosis, necrosis, and pyroptosis (13, 14). Given the complexity of autoimmune diseases, a single type of PCD seems inadequate to explain disease progression. Consequently, the role of PANoptosis, which integrates features of these PCDs, has gained attention. Emerging studies suggest that PANoptosis may act as a key regulator in autoimmune diseases (15, 16). This review examines the mechanisms of PANoptosis activation and maintenance, as well as its connection to autoimmune diseases. Our goal is to provide a comprehensive understanding of PANoptosis in autoimmune diseases to identify new therapeutic targets and strategies for better disease management.

## 2 Overview of PANoptosis

Apoptosis, necrosis, and pyroptosis are three well-established types of PCD. Initially thought to operate independently, research now shows extensive crosstalk and connections among these types as our understanding of PCD deepens (17, 18). For example, caspase-8, traditionally a key regulator of apoptosis, also mediates pyroptosis by modulating the NLRP3 inflammasome or cleaving gasdermin D (GSDMD) directly (19). Similarly, caspase-3, a key regulator of apoptosis, can induce mitochondrial damage and secondary necrosis by cleaving BH3 interacting domain death agonist (BID) without GSDMD (20). PANoptosis incorporates key components from apoptosis, necrosis, and pyroptosis. It is regulated by a cascade of signaling molecules triggered by pattern recognition receptors (PRRs) that detect pathogen-associated molecular patterns (PAMPs) and damage-associated molecular patterns (DAMPs). This initiates the assembly of the PANoptosome complex, activating downstream execution molecules, including caspase-3/7, GSDMD, gasdermin E (GSDME), and Mixed Lineage Kinase Domain-Like (MLKL), leading to membrane perforation and cell lysis (21). The concept of PANoptosis highlights the coordination and crosstalk among apoptosis, necrosis, and pyroptosis in response to pathogens and innate immune triggers, addressing a gap in understanding the interactions between molecular components of distinct PCDs (22).

## 3 The composition and regulatory mechanisms of the PANoptosome

**Figure 1:**
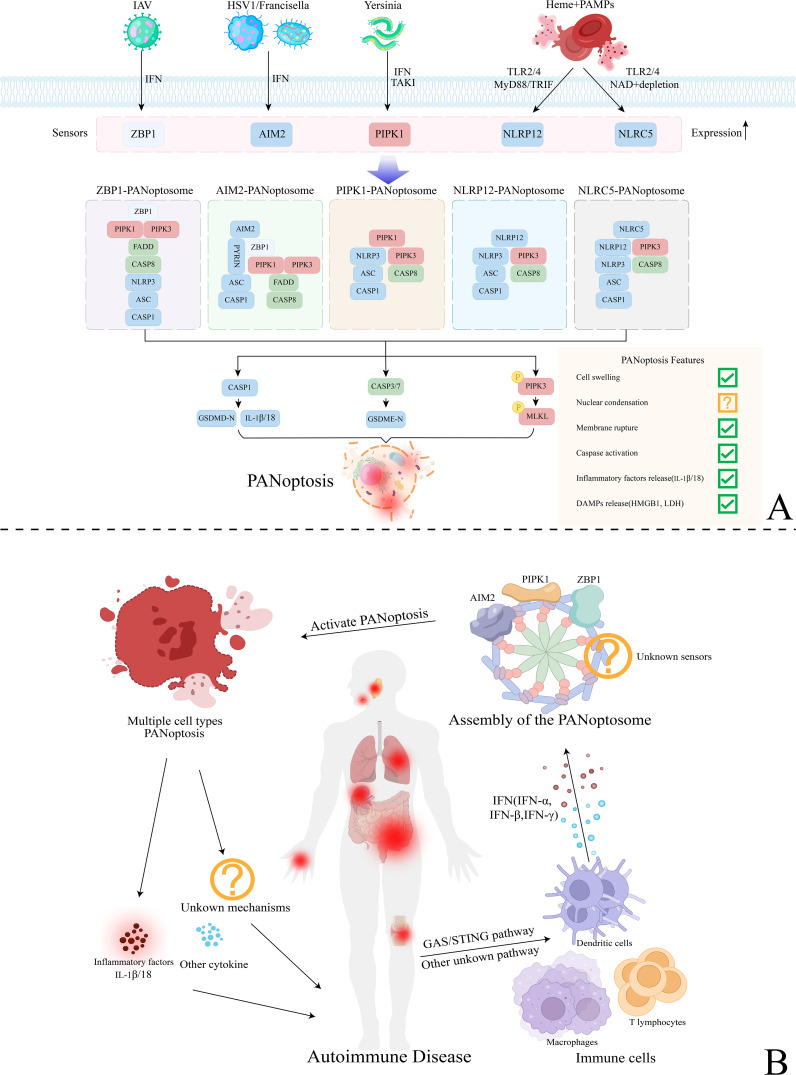
Mechanisms of PANoptosis activation and the regulatory mechanisms of PANoptosis in autoimmune diseases. **(A)** Various mechanisms of PANoptosis activation mediated by different types of PANoptosomes. Sensor molecules such as ZBP1, AIM2, PIPK1, NLRP12, and NLRC5 detect pathogens like IAV, HSV, Yersinia, and heme, regulated by upstream mechanisms like TLR2/4 and IFN. Subsequently, they assemble the PANoptosome by integrating key components from other programmed cell death (PCD) pathways with the help of adapters. This process ultimately activates downstream effector molecules, leading to GSDMD-mediated pyroptosis, Caspase-3/7-mediated apoptosis, and MLKL-mediated necrosis (PANoptosis). **(B)** The regulatory mechanisms of PANoptosis in autoimmune diseases. Various immune cells, including macrophages, dendritic cells, and T lymphocytes, regulate upstream pathways like GAS/STING, resulting in the release of significant amounts of IFN (IFN-α, IFN-β, IFN-γ). This process activates PANoptosome assembly and inducing PANoptosis in various cell types. PANoptosis may exacerbate systemic inflammatory responses and tissue damage associated with autoimmune diseases by releasing inflammatory factors like IL-1β/18 and harmful cytokines. Additionally, it may affect the onset and progression of the disease through other potential pathways, warranting further investigation.

The assembly of the PANoptosome is a critical step in PANoptosis. It integrates core components from apoptosis, necrosis, and pyroptosis pathways, such as inflammasomes, death-inducing signaling complexes (DISC), and necrosomes (23). PANoptosome proteins can be categorized into three functional groups: sensors that respond to stimuli, effectors that execute functions, and adapters that link sensors to effectors (24).To respond to diverse stimuli, PANoptosomes with varying sensors and effectors are required to initiate PANoptosis. These PANoptosomes are distinguished by their composition and the downstream molecular mechanisms they activate (23). So far, several established PANoptosomes include Z-DNA binding protein 1 (ZBP1), Absent in Melanoma 2 (AIM2), Receptor-Interacting Protein Kinase 1 (RIPK1), NOD-like receptor family pyrin domain-containing 12 (NLRP12), and NOD-like receptor family CARD domain-containing 5 (NLRC5)-PANoptosomes. Furthermore, interferon (IFN) signaling serves as a key upstream regulator of PANoptosome assembly. For example, IFN regulatory factor 1 (IRF1) regulates the expression of several PANoptosis sensor molecules (25). Studies show that blocking IFN-α, IFN-β, or IRF1 to inhibit IFN signaling can suppress ZBP1 expression induced by influenza A virus (IAV) (26) (**Figure 1A**).

### 3.1 ZBP1-PANoptosome

The ZBP1-PANoptosome was first identified during IAV infection, comprising the primary sensor ZBP1, secondary sensor NLRP3, adapters Fas-associated protein with a death domain (FADD) and apoptosis-associated speck-like protein containing a CARD (ASC), along with effectors Caspase-1/6/8, RIPK1, and receptor-interacting protein kinase 3 (RIPK3) (27). ZBP1 has two Z-nucleic acid binding domains (Zα1 and Zα2) at its N-terminus, where the Zα2 domain recognizes viral infections (28). In the early stages of IAV infection, innate immune sensing of viral RNA activates ZBP1, initiating the assembly of the ZBP1-PANoptosome and triggering PANoptosis. ZBP1 interacts with RIPK3 through its RHIM domain, activating caspase-8 and promoting MLKL phosphorylation, which triggers both apoptosis and necroptosis pathways. Simultaneously, ZBP1 interacts with NLRP3 through the FADD- and caspase-8-containing DISC, leading to the release of interleukin (IL)-1β and IL-18, thereby activating pyroptosis pathway (29).

### 3.2 AIM2-PANoptosome

AIM2 is a cytosolic pattern recognition receptor (PRR) that detects pathogens and endogenous double-stranded DNA (dsDNA) (30). Previous studies have demonstrated that AIM2, as an inflammasome sensor, binds cytosolic dsDNA from pathogens through its C-terminal HIN-200 domain and interacts with ASC via its N-terminal pyrin domain, promoting inflammasome assembly and inducing pyroptosis (31). However, a recent study revealed that during Herpes Simplex Virus Type 1 (HSV-1) and F. novicida infections, AIM2 interacts with ZBP1 and Pyrin to form the AIM2-PANoptosome, inducing PANoptosis (32). Given AIM2’s role as an inflammasome sensor, investigating the components and mechanisms of the AIM2-PANoptosome is crucial for understanding innate immunity, inflammation, and cell death.

### 3.3 RIPK1–PANoptosome

Malireddi et al. (33) discovered that Yersinia induces RIPK1-independent PANoptosis. Immunoprecipitation revealed that RIPK1 co-precipitates with RIPK3, caspase-8, ASC, FADD, and NLRP3 during Yersinia infection, suggesting that RIPK1 may form a PANoptosome with these key cell death components. The toxin YopJ, produced by Yersinia, activates RIPK1 and drives cell death by inhibiting transforming growth factor β-activated kinase 1 (TAK1) (34). TAK1 serves as a major negative regulator of RIPK1-mediated PANoptosis, preventing spontaneous pyroptosis, apoptosis, and necroptosis by inhibiting RIPK1 phosphorylation (35). However, studies show that knocking out RIPK1 during Yersinia infection does not fully prevent cell death. This could be due to ZBP1 activation in the absence of RIPK1 or the initiation of other compensatory mechanisms that promote cell death (9). Currently, studies have only demonstrated the TAK1-regulated activation and assembly mechanism of the RIPK1-PANoptosome in Yersinia infection. Further research is needed to clarify its regulatory mechanisms in other disease contexts.

### 3.4 NLRP12-PANoptosome

NLRP12, a member of the NOD-like receptor (NLR) family, is closely linked to innate immunity and inflammatory diseases (36). Previous studies have demonstrated that during Yersinia pestis or P. chabaudi infections, NLRP12 functions as a cytosolic PRR, serving as an inflammasome sensor to drive pyroptosis (36, 37). Besides activating the inflammasome, recent studies suggest that under co-stimulation with heme and PAMPs, NLRP12 can also act as a PANoptosome sensor, driving PANoptosis (38). In this process, upstream Toll-like receptors (TLR) 2 and 4 regulate NLRP12 expression via IRF1.

### 3.5 NLRC5-PANoptosome

NLRC5, a member of the NLR family, has primarily been studied for its role in regulating the expression of Major Histocompatibility Complex Class I (MHC I) genes (39). However, its role in regulating innate immunity, inflammation, and cell death remains largely unexplored. A recent study revealed that under stimulation by heme and PAMPs, NLRC5 interacts with NLRP12 and other cell death molecules to activate the TLR and NAD+-dependent assembly of the NLRC5-PANoptosome, inducing PANoptosis (40). Although this study identified the NLRC5-PANoptosome under heme and PAMP stimulation, the direct interactions among its complex components need further characterization.

### 3.6 Caspase family

The caspase family is a core regulator of apoptosis, necrosis, and pyroptosis, serving as a critical component of PANoptosis and playing essential roles throughout the process. Caspase-1/3/7 serve as downstream executioners in PANoptosis, responding to PANoptosome assembly and ultimately leading to cell death. Caspase-8 is a key effector in PANoptosomes, regulating the interaction and balance among the three cell death pathways in PANoptosis (41).During apoptosis, caspase-8 is activated by the DISC, which contains FADD and RIPK1. Caspase-8 then cleaves and inactivates RIPK1, preventing necrosome formation (42). When caspase-8 is inhibited, RIPK1 forms a necrosome with RIPK3 and FADD, triggering necrosis (9). Additionally, the RIPK1/FADD/caspase-8 complex can directly activate the NLRP3 inflammasome, releasing caspase-1, which leads to GSDMD pore formation and the release of IL-1β and IL-18 (43). A recent study found that caspase-6, a key apoptotic effector, acts as a scaffold to enhance RHIM domain-dependent interactions between RIPK3 and ZBP1, promoting PANoptosome assembly (44).

## 4 Dual role of PANoptosis in diseases and therapeutic implications

Research suggests that PANoptosis, an integrated form of necrosis, pyroptosis, and apoptosis, plays a crucial dual role in various diseases. In microbial infections, ZBP1-induced PANoptosis and cytokine storms raise mortality in COVID-19 patients (45), whereas AIM2-induced PANoptosis enhances host defense against HSV1 or F. novicida infections (32). In tumors, studies indicate that high expression of PANoptosis-related genes harms low-grade glioma (LGG) and kidney renal cell carcinoma (KIRC), but benefits skin melanoma (SKCM) (46). PANoptosis can also worsen inflammation and organ damage in diseases. The stimulator of IFN genes (STING) agonist diABZI triggers PANoptosis, causing inflammation and acute respiratory distress syndrome (ARDS) (47). Uysal E et al. showed that inhibiting PANoptosis specifically protects rats from kidney ischemia-reperfusion injury (48). Given PANoptosis’s dual role in diseases, endogenous molecules or compounds targeting its inhibition or promotion have shown therapeutic potential across various conditions (49). The endogenous molecule DKK1 reduces diabetic retinopathy in rats by inhibiting PANoptosis (50), whereas CurE promotes ZBP1-dependent PANoptosis to suppress ACC tumor growth in nude mice (51); miR-29a-3p agomir injection therapy reduces lung damage in mice with acute lung injury (ALI) by inhibiting PANoptosis in alveolar epithelial cells (52). Although PANoptosis modulators show promising therapeutic potential, current strategies have only been tested *in vitro* and in animal models, and the relationship between PANoptosis and diseases remains unclear. Further research into the specificity, efficacy, and safety of PANoptosis modulators is needed.

## 5 The potential mechanisms of PANoptosis in autoimmune diseases

Rheumatoid Arthritis (RA), Systemic Lupus Erythematosus (SLE), Sjögren’s Syndrome (SS), and several other autoimmune diseases, including Psoriasis, Ulcerative Colitis(UC), and Crohn’s disease(CD), have been confirmed to be associated with PANoptosis. Here, we summarize the evidence for PANoptosis occurrence in various autoimmune diseases (**Table 1**) and explore the potential mechanisms underlying PANoptosis activation and regulation (**Figure 1B**).

**Table 1:** Evidence for the occurrence of PANoptosis in autoimmune diseases.

Autoimmune Disease	Study type	Analytical technique	Result	Reference
SLE	Peripheral blood samples/PBMCs from SLE patients	Bioinformatics analysis and validation based on GeneCard database and GEO database	Five key PANoptosis signature genes were identified and validated, including ZBP1, MEFV, LCN2, IFI27, HSP90AB1	(53)
RA	Synovium in patients with RA	Bioinformatics analysis based on GeneCard database and GEO database	Identified the PANoptosis biomarker SPP1, discriminated between two distinct RA subtypes, and developed a scoring model with potential in distinguishing subtypes	(54)
SS	NSG mice transplanted with PBMCs from SS patients	Expression of type I IFN and PANoptosis-related genes in submandibular glands of NSG mice transplanted with PBMCs from SS patients detected by laboratory techniques	IFN signaling activation and PANoptosis characterized genes	(55)
psoriasis	Lesion skin samples	Bioinformatics analysis based on GeneCard database and GEO database	Ten PANoptosis-related hub genes were identified, namely AIM2, BAK1, CASP1, CASP4, CASP5, GZMA, GZMB, IL18, IRF1, PYCARD	(56)
psoriasis	Lesion skin samples/spleen and skin samples from psoriasis mice	Bioinformatics analysis and validation based on MSigDB database and single-cell dataset	PANoptosis-related hub genes (S100A12, CYCS, NOD2, STAT1, HSPA4, AIMAM2, MAPK7) were identified	(57)
DC	DC patient intestinal mucosa/DC mouse	Bioinformatics analysis and validation based on GeneCard database and GEO database	Ten PANoptosis-associated core genes were identified and validated, and two PANoptosis clusters with unique immune penetration and functional patterns were identified	(58)
UC	Intestinal mucosa of UC patients/UC mice	Bioinformatics analysis and validation based on GeneCard database and single-cell datasets	Identification of key genes associated with autophagy in PANoptosis	(59)
UC	Intestinal mucosa of UC patients/UC mice	Bioinformatics analysis and validation based on GEO database	Five key genes for PANoptosis were identified (ZBP1, AIM2, CASP1/8, IRF1), a ceRNA network for the key genes was established, and three potential small molecule drugs were obtained	(60)

### 5.1 SLE and PANoptosis

SLE is a complex autoimmune disease marked by a loss of tolerance to self-antigens, leading to autoantibody production. This overactive immune system causes immune complexes to deposit in tissues and organs, leading to damage (61, 62). The pathogenesis of SLE is multifactorial, involving genetics, sex, and environmental factors.Among these, IFN signaling is central to SLE pathogenesis (63, 64). Plasmacytoid dendritic cells (pDCs) synthesize Type I IFN, especially IFN-α, the predominant IFN in SLE. Other cell types, like macrophages and fibroblasts, primarily produce IFN-β (65, 66). Type II IFN also significantly contribute to SLE. Studies show that PBMCs from SLE patients have higher levels of IFN-γ and IRF1 than those from healthy individuals. Abnormal IFN-γ accumulation can be detected early in SLE, even before autoantibodies or Type I IFN appear (67, 68). Continuous IFN production worsens the immune response, promoting immune cell proliferation and differentiation while activating B cells to produce autoantibodies, leading to complications like lupus nephritis (LN) (69). Previous studies show various types of PCD occur in SLE. For instance, NLRP3 expression correlates with disease activity in SLE patients and is upregulated in macrophages (13, 70); neutrophil apoptosis increases in SLE (71); MLKL, a key necroptosis protein, is elevated in SLE PBMCs (72). Multiple PCD types can also occur within the same cell type in SLE. Guo et al. (73) found that both necroptosis and pyroptosis are activated in podocytes from LN patients and lupus mice kidneys. Bioinformatics analyses identify key PANoptosis-related genes like ZBP1, AIM2 and NLRP3 as biomarkers for SLE (53, 74, 75). All studies identified ZBP1 as a diagnostic biomarker for SLE. This suggests that in SLE, these cell death processes may interact via ZBP1-mediated PANoptosis to influence disease progression. As mentioned, IFN signaling is a key regulator of PANoptosome assembly. Chronic inflammation in SLE causes continuous accumulation of mitochondrial reactive oxygen species (ROS), with mitochondrial DNA (mtDNA) released as DAMPs (76, 77). DNA sensors like cyclic GMP-AMP synthase (cGAS) and ZBP1 trigger the release of large amounts of IFNs (78). These IFNs upregulate ZBP1 expression, promoting ZBP1-PANoptosome assembly and inducing PANoptosis. This may indicate a potential PANoptosis mechanism in SLE. In conclusion, the relationship between SLE and PANoptosis is still unclear. Further studies are needed to identify which PANoptosomes mediate PANoptosis in specific cell types and how PANoptosis influences disease progression.

### 5.2 RA and PANoptosis

RA is a chronic autoimmune disease that can progressively damage tissues and organs beyond the joints, affecting the heart, liver, kidneys, and skin (79, 80). RA is associated with environmental, gender, and genetic factors. The immune system mistakenly attacks the joints, leading to inflammation and damage (81, 82). Recent research shows complex connections between cell death and RA. For instance, fibroblast-like synoviocytes (FLS) in RA resist apoptosis, leading to synovial proliferation and inflammation (83). A cohort study indicates that the percentage of granulocytes undergoing apoptosis and primary necrosis is significantly higher in RA patients than in healthy controls (84). Pyroptosis has also been observed in FLS, monocytes, and macrophages from RA patients (85). RA involves crosstalk among various programmed cell death (PCD) types across different cell populations, regulating inflammation and immune responses. One type of PCD alone is insufficient to explain the progression of RA. Studies show that several PANoptosis-related molecules are significantly upregulated in different RA cell types. For example, NLRP3 and GSDME are increased in FLS (86, 87). Caspase-3 and GSDME are upregulated in RA monocytes and macrophages (88), and neutrophils in RA joints exhibit CD44 and GM-CSF-dependent necrosis with increased RIPK1, RIPK3, and MLKL (89). This suggests that PANoptosis may occur in various cell populations in RA. A meta-analysis found higher levels of the PANoptosis sensor AIM2 in PBMCs from RA patients (90). Two bioinformatics analyses identified AIM2 as a key gene for RA (91, 92). Chen et al. (93) detected higher levels of AIM2 and the adapter ASC in RA patients’ synovial tissues using immunohistochemistry. Introducing AIM2 siRNA into FLS significantly inhibited their proliferation. This indicates that PANoptosis in RA may involve the AIM2-PANoptosome. However, the specific cell types involved and the upstream regulatory mechanisms are still unclear and need further investigation.

### 5.3 SS and PANoptosis

SS is a systemic autoimmune and lymphoproliferative disorder characterized by uncontrolled lymphoplasmacytic infiltration in exocrine glands, such as the salivary and lacrimal glands, resulting in inflammation and tissue damage. Abnormally proliferating lymphocytes can also damage other organs, including the lungs, kidneys, and blood vessel walls (94, 95). IFN is also a key pathogenic factor in SS. Multiple studies have shown that IFN response genes are upregulated in various cell types in SS, and inhibiting IFN signaling can effectively alleviate SS progression (96). In SS, IFN production stems from stimulation of the DNA sensing pathway. Damaged genomic DNA accumulates in the cytoplasm, activating the DNA sensor cGAS, which produces cyclic GMP-AMP (cGAMP) and activates the STING, inducing Type I IFN production (97, 98). Previous studies have shown that the expression of multiple PANoptosis components related to apoptosis, pyroptosis, and necroptosis is upregulated in SS. For instance, Type I IFN increases the expression of key pyroptosis proteins such as AIM2, ASC, caspase-1, and GSDMD in the salivary gland epithelial cells (SGECs) of SS patients (99). In SGECs of SS patients, the expression of apoptosis-related protein caspase-8 and necroptosis markers p-MLKL and RIPK3 is also upregulated (100); in NSG mouse models transplanted with PBMCs from SS patients, submandibular glands exhibited upregulation of Type I IFN and genes related to PANoptosis (necroptosis and apoptosis) (55, 101). This evidence suggests that these key PCD components may integrate into a larger PANoptosome in SS, influencing disease progression. Additionally, studies have revealed abnormal cytoplasmic accumulation of damaged genomic DNA in SS patient samples, with impaired DNase1 expression and activity in the SGECs and ductal tissues of SS patients (102). AIM2 is a typical cytosolic DNA sensor activated by accumulated genomic DNA in the cytoplasm (103). Vakrakou et al. (102) suggest that the intrinsic activation of ductal epithelial cells in SS patients is caused by the sustained activation of AIM2 due to the accumulation of cytoplasmic DNA. This suggests that in SS, AIM2, with the assistance of IFN signaling, may recognize abnormally accumulated cytoplasmic DNA, assemble the AIM2-PANoptosome, and trigger PANoptosis in multiple tissues. Furthermore, a study has shown that mitochondrial damage in SS leads to the release of mtDNA into the cytoplasm, which, like cytoplasmic DNA, may serve as a potential pathway for activating the AIM2-PANoptosome (104).

### 5.4 Other autoimmune diseases and PANoptosis

Different types of cell death occur in autoimmune diseases like psoriasis, UC, and CD (105–107). Similarly, IFN is a key pathogenic factor in these diseases. Research indicates that sustained IFN-γ release characterizes psoriasis (108), while the joint disruption of intestinal epithelial barriers by IFN-γ and TNF-α is crucial in UC and CD (109). This suggests that IFN may induce PANoptosis in these diseases through the activation of various sensor molecules. Several studies have demonstrated that PANoptosis sensor molecules and key components are activated in these diseases (110, 111). The inflammasome sensor AIM2 has been identified as a susceptibility gene locus for psoriasis, closely associated with the genetic and epigenetic factors of the disease, with increased expression in psoriatic keratinocytes (112); a recent study found significant upregulation of DNA sensors ZBP1, the NLRP3 inflammasome, and cGAS/STING, along with IFN signaling molecules (IFN-β, IFN-γ, and TNF-α) in colonocytes from active UC patients (98). Furthermore, multiple bioinformatics analyses identified PANoptosis-related genes as diagnostic markers for these diseases, confirming the involvement of PANoptosis and highlighting its potential as a therapeutic target (56, 58–60). For example, one study identified 10 PANoptosis-related core genes in psoriasis by analyzing 33 skin samples across three datasets. Immune infiltration analysis indicated that PANoptosis might influence psoriasis progression by regulating M1 and M2 macrophage polarization. Another study identified 10 PANoptosis-related core genes in 279 CD samples and created gene-miRNA, gene-transcription factor, and drug-gene interaction networks, revealing two distinct PAN clusters with unique immune infiltration and functional patterns. Although evidence suggests the potential for IFN-mediated PANoptosis in these autoimmune diseases, further experiments, including Co-IP, are necessary to confirm the presence of PANoptosis and clarify the specific cell types and mechanisms involved.

## 6 Discussion and perspectives

PANoptosis, a newly identified PCD form, highlights the crosstalk and redundancy among PCDs, providing a deeper understanding of the link between innate immunity and PCD. As a unique innate immune-inflammatory PCD pathway, PANoptosis has significant pathophysiological relevance to infections, autoimmune diseases, and inflammation. Consequently, the link between autoimmune diseases and PANoptosis represents an emerging and highly promising research area. Moreover, several key components of PANoptosis have been identified in various autoimmune diseases and implicated in their development. This suggests PANoptosis could be a novel therapeutic target in autoimmune diseases.

Although PANoptosis shows great potential in autoimmune diseases, like many emerging fields, research on PANoptosis in this area faces numerous unresolved challenges. First, the structure and activation mechanisms of the PANoptosome in autoimmune diseases remain poorly understood. Validating the structure and regulatory mechanisms of the PANoptosome in any single disease is challenging. Further characterization of the PANoptosome’s structure and upstream regulatory mechanisms in autoimmune diseases is essential. Secondly, the role of PANoptosis in autoimmune diseases remains unclear, and most therapeutic strategies targeting it have only been validated in other disease models. Given the dual role of PANoptosis in various diseases, it may similarly affect autoimmune diseases. In RA, PANoptosis of FLS may reduce synovial proliferation, while inflammatory factors produced by PANoptosis could worsen inflammation and tissue damage. However, the role of PANoptosis in autoimmune diseases is both complex and unclear. Further research is required to explore overall role of PANoptosis in autoimmune diseases, as well as its specific roles in different disease stages and cell types, to develop safe and effective therapeutic strategies.
